# A Fast kNN Algorithm Using Multiple Space-Filling Curves

**DOI:** 10.3390/e24060767

**Published:** 2022-05-30

**Authors:** Konstantin Barkalov, Anton Shtanyuk, Alexander Sysoyev

**Affiliations:** Department of Mathematical Software and Supercomputing Technologies, Lobachevsky University, 603950 Nizhny Novgorod, Russia; anton.shtanyuk@itmm.unn.ru (A.S.); alexander.sysoyev@itmm.unn.ru (A.S.)

**Keywords:** machine learning, kNN, dimensionality reduction, multiple space-filling curves

## Abstract

The paper considers a time-efficient implementation of the *k* nearest neighbours (kNN) algorithm. A well-known approach for accelerating the kNN algorithm is to utilise dimensionality reduction methods based on the use of space-filling curves. In this paper, we take this approach further and propose an algorithm that employs multiple space-filling curves and is faster (with comparable quality) compared with the kNN algorithm, which uses kd-trees to determine the nearest neighbours. A specific method for constructing multiple Peano curves is outlined, and statements are given about the preservation of object proximity information in the course of dimensionality reduction. An experimental comparison with known kNN implementations using kd-trees was performed using test and real-life data.

## 1. Introduction

Currently, machine learning (ML) methods are being used successfully to solve a wide range of problems in various application areas. One example of a class of problems where ML has demonstrated its effectiveness are the tasks of identifying the main properties of the phenomena, which are characterised by their stochastic nature or the presence of hidden parameters [1,2,3].

In many cases, solving application problems comes down to the problem of classification, i.e., assigning the object under study to one of the available classes. One of the well-known classification methods is the kNN (*k* nearest neighbours) method. Although the earliest papers related to this method appeared more than 50 years ago [4,5], theoretical studies of various aspects of using kNN are still ongoing [6,7]. This is not to mention hundreds of publications in which this method has been used to solve applied classification problems. Over many years of use, kNN has established itself as a simple and reliable method that yields acceptable results when solving many different problems.

Among the undoubted merits of kNN is its “explainability”, since the decision about whether a test object belongs to one class or another is clearly explained by similar properties of the test object and its nearest (in some metric) neighbours. Along with the study of the method’s theoretical properties, the issue of its effective implementation is also investigated. Thus, direct implementation of the method by using the “brute-force” approach has computational complexity O(n), where *n* is the number of objects. Fast algorithms, which have computational complexity O(log(n)), use complex data structures of kd-tree type [8].

One approach to accelerating the kNN algorithm is to use dimensionality reduction methods involving space-filling curves (Peano–Hilbert curves) [9,10]. In this paper, we develop this approach further and propose an algorithm that employs multiple space-filling curves and is faster (with comparable quality) compared with the algorithm using kd-trees. A specific method for constructing multiple Peano curves is outlined, and statements are given about the preservation of object proximity information in the course of dimensionality reduction. Comparison with known kNN implementations (in particular, those using kd-trees) was performed using test and real-life data with small dimensionality, i.e., just those data for which kd-trees show the best performance in terms of speed.

The paper is organised as follows. Section 2 contains the problem statement and description of the kNN method. Section 3 describes the scheme for constructing a Peano–Hilbert curve approximation (evolvent) and the kNN method, which uses the evolvent to reduce the dimensionality of the data. The fundamental drawbacks of this dimensionality reduction method are noted. Section 4 proposes a way to overcome the drawbacks mentioned, based on the use of multiple evolvents. A theoretical statement is formulated about preserving some proximity information in the multidimensional space on one of the one-dimensional scales. A kNN scheme using multiple evolvents is presented in Section 5. Section 6 contains experimental results comparing different implementations of kNN using synthetic and real-life data. Section 7 concludes the paper.

## 2. Problem Statement

In this study, the classification problem will be understood as the problem of assigning an object ω to one of the predetermined classes based on its features. We will assume that each of the objects ω is represented as a feature vector

(1) $$y=\left(y_{1},y_{2},...,y_{N}\right)\in R^{N},$$

where the value of the *j*-th vector coordinate corresponds to the numerical value of the *j*-th feature of the object ω. We will also assume that there is already some number of objects exactly classified (a test sample), i.e., for each object, we know which class it belongs to.

The *k* nearest neighbours (kNN) method is based on the following simple rule: an object is considered to belong to the class to which most of its nearest neighbours belong. Here, “neighbours” refers to objects that are close (in one sense or another) to the one being studied. Note that in order to apply this method, some metric $L(\omega_{i},\omega_{j})$—i.e., the distance function—should be introduced in the feature space of objects. As a rule, the Euclidean distance is employed here, although other metrics may also be used [7].

The general scheme of object classification using kNN can be formulated as follows:

- Calculate the distance from the object being classified to each of the objects in the training set;
- Select *k* objects of the training set with the minimum distance to them;
- The class of the object being classified is the class most often found among the *k* nearest neighbours.

In the practical implementation of kNN, an important indicator is the estimate of the time complexity of the neighbour finding procedure. The algorithms and data structures on which the search procedure is based include, in particular, the following.

- Brute force method. This is based on calculating all distances from the test object to other objects of the class and determining the smallest value. This method has complexity O(n), where *n* is the number of objects.
- KD-tree method. This is based on a special kind of binary tree, where each node represents a point in the multidimensional space. The search procedure has complexity O(log(n)).
- Ball-tree method. This is yet another kind of tree structure, which has logarithmic complexity. This method is applicable to problems that have a large dimensionality.

The main drawback of the brute force method is the unacceptable running time and the rapid growth of the computation volume as the number of objects increases. This method can be applied when the number of objects is relatively small and the dimension of *y* is small, and when the “curse of dimensionality” is not yet in full effect.

For kd-tree, the main drawback is that it slows down when the number of objects grows, which is caused by increasing complexity of the internal tree structure. However, kd-trees have proven to be a good solution for problems with small dimensionality, which will be considered in this study.

As mentioned, the computational cost of searching for nearest objects increases with increasing dimensionality, both in the case of exhaustive search for all distances and when using tree structures. One mechanism for speeding up the search is dimensionality reduction—by reducing the problem of searching in a multidimensional space to the search in a one-dimensional space (over an interval). This is possible through the use of space-filling curves (Peano–Hilbert curves) that fill the multidimensional space. Such curves are used, for example, in global optimisation [11,12,13,14], in numerical approximation of solutions to systems of nonlinear inequalities [15], in image processing [16,17,18], etc.

We should also note that in addition to space-filling curves, there are other similar curves, such as area-filling curves [19]. However, it is the Peano–Hilbert curves that are commonly used due to the relative simplicity of their construction and a number of useful properties, of which the key ones will be discussed in Section 3 and Section 4.

An algorithm based on data curves can offer some advantages over tree-based algorithms by reducing the search time. Neighbour detection for test objects includes two phases: building and initialising the data structures (build step) and performing a search or query (query step). In most cases, the build phase occurs only once, while queries are repeated many times and can significantly affect the overall speed of the classification algorithm.

Note that in practical classification tasks, different features can have different scales, which can significantly distort the real distance between objects. Therefore, it will be assumed that prior to applying kNN, data scaling has been performed

(2) $$y_{j}=\frac{y_{j}-y_{min}}{y_{max}-y_{min}}-\frac{1}{2}.$$

Thus, the variation domain for all feature values will form a unit hypercube

(3) $$D=\{y\in R^{N}:-1/2\leq y_{j}\leq 1/2,1\leq j\leq N\}.$$

The 1/2 hypercube offset has been made for the convenience of further labelling.

## 3. Dimensionality Reduction Using Space-Filling Curves

Let us briefly describe a general scheme for constructing a space-filling curve. We will consider here Hilbert’s scheme for constructing such a curve.

(1). Divide hypercube *D* form (3) with edge length of 1 by coordinate hyperplanes into $2^{N}$ hypercubes of the first partition (with edge length of 1/2).

Then, divide every hypercube of the first partition into $2^{N}$ hypercubes of the second partition (with edge length of 1/4) by hyperplanes parallel to coordinate hyperplanes and passing through midpoints of hypercube edges orthogonal to them.

By continuing the above process, i.e., by sequentially partitioning each subcube of the current partition into $2^{N}$ of the next partition, we can construct hypercubes of any *M*-th partition with edge length of ${\left(1/2\right)}^{M}$. The total number of subcubes of the *M*-th partition will be equal to $2^{NM}$.

(2). Now, divide the segment [0,1] into $2^{N}$ equal parts, divide each of them also into $2^{N}$ equal parts and so on. Denote the subinterval of the *M*-th partition by d(M,v), where *v* is the coordinate of the left boundary point of the subinterval. Obviously, the length of the subinterval d(M,v) will be equal to $2^{-NM}$. We will assume that the left boundary point belongs to the subinterval and the right boundary point does not. The only exception is the subinterval whose right boundary point is 1, in which case it also belongs to the subinterval.

(3). Establish a one-to-one correspondence between subintervals and subcubes of the *M*-th partition. We denote by D(M,v) a subcube corresponding to the subinterval d(M,v). This correspondence should satisfy the following requirements.

Condition 1. $D\left(M+1,v^{\prime }\right)\in D\left(M,v^{\prime \prime }\right)$ i.f.f. $d\left(M+1,v^{\prime }\right)\in d\left(M,v^{\prime \prime }\right)$.

Condition 2. Two subintervals $d(M,v^{\prime })$ and $d(M,v^{\prime \prime })$ share a common endpoint i.f.f. the corresponding subcubes $D(M,v^{\prime })$ and $D(M,v^{\prime \prime })$ are contiguous, i.e., share a common edge.

Condition 3. If x∈d(M,v) then y(x)∈D(M,v),M≥1.

Note that the centre $y^{c}\left(x\right)$ of the *M*-th partition subcube containing point y(x) can be regarded as an approximation to y(x) with the accuracy of $\varepsilon =2^{-(M+1)}$, i.e.,

(4) $$\max_{1\leq j\leq N}\left|y_{j}^{c}\left(x\right)-y_{j}\left(x\right)\right|\leq \varepsilon =2^{-(M+1)}.$$

The function $y^{c}\left(x\right)$ maps a uniform grid with step $2^{-NM}$, constructed in the interval [0,1], onto a uniform grid with step $2^{-M}$, constructed in the hypercube *D*. A constructive way of establishing such a correspondence is described and theoretically justified in [20,21]. The algorithmic complexity of computing the value of the function $y^{c}\left(x\right)$ depends on the dimensionality *N* on the partitioning level *M* and is O(M·N).

Using the Peano–Hilbert curve, one can propose the following scheme for implementing the kNN algorithm.

Let $\omega_{i},1\leq i\leq S$ be the *i*-th object from the set of objects correlated with class $C_{j}$. Previously, a feature vector $y=\left(y_{1},y_{2},...,y_{N}\right)\in R^{N}$ corresponding to each object under consideration was defined, i.e., 

$$\omega_{i}↔y=\left(y_{i1},y_{i2},...,y_{iN}\right).$$

Using the Peano curve, each object is assigned the value of $x_{i}$, i.e.,

$$\omega_{i}↔x_{i},\;y\left(x_{i}\right)=\left(y_{i1},y_{i2},...,y_{iN}\right).$$

Consider a test object $\omega^{*}$, to which the value $x^{*}$ is assigned. The degree of proximity of the test object $\omega^{*}$ to one of the objects $\omega_{i}$ is determined based on the distance on the one-dimensional scale $d_{i}=\left|x_{i}-x^{*}\right|$. Thus, the closest object to the test object has the following property:

$$d_{min}=\min\left\{d_{i}:1\leq i\leq S\right\}.$$

The operation of the algorithm to determine the nearest neighbour of the test object involves two phases: data preparation and the search itself. Data preparation consists of calculating the correspondence $\omega_{i}\to x_{i}$ for each input object, including the test object, and ordering the objects by the value of *x*.

$$x^{1}\left(\omega^{1}\right)\leq x^{2}\left(\omega^{2}\right)\leq ...\leq x^{S}\left(\omega^{S}\right).$$

Since the corresponding object preimages $x_{i}\in \left[0,1\right]$ are ordered, the closest object can be found using a fast binary search algorithm.

When *K* neighbours need to be found, this search procedure can be repeated *K* times. In this case, it is necessary to exclude previously found items from the search. This can be achieved by excluding $x_{i}$ from the set being searched or by comparing the found $x_{i}$ with the previously found ones to find a match. For example, one possible implementation of searching for *K* nearest neighbours could be as follows: in relation to $x^{*}$, we look for the nearest values to the left and right on the one-dimensional scale, choose the minimum one and continue searching sideways until exactly *K* neighbours are found.

Note that the method based on space-filling curves can be applied not only to numerical features but also to categorical features. The values of categorical features are discrete, so this approach requires a numerical value—e.g., from the interval [0, 1]—to be assigned to each discrete value. A specific method of assigning numerical values to categorical variables is described in Section 6.2 for the CarEvaluation dataset.

One obvious drawback of using the Peano curve in the kNN method is the loss of much of the information about the proximity of objects in multidimensional space when constructing their preimages on the one-dimensional scale.

**Figure 1:**
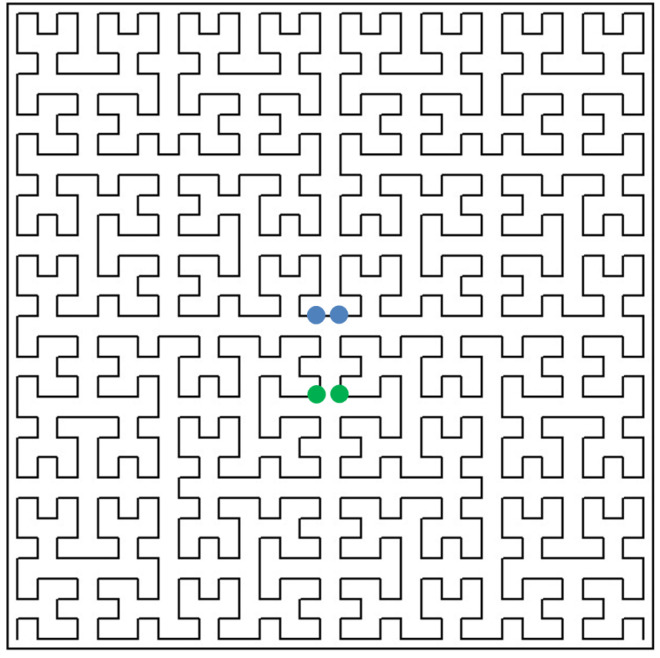
Peano curve.

Indeed, the point x∈[0,1] on the one-dimensional scale has only left and right neighbours, whereas its corresponding object $y\left(x\right)\in R^{N}$ can have neighbours along *N* coordinate directions. As a result, when using Peano-curve-type mappings, close objects $y^{\prime }$, $y^{\prime \prime }$ in the *N*-dimensional space can have their quite distant corresponding preimages $x^{\prime }$, $x^{\prime \prime }$ on the interval [0,1]. In Figure 1, the green dots show two objects that are close in the two-dimensional space, while their preimages on the one-dimensional scale are far away from each other. The blue dots correspond to two objects, the distance between which is retained when changing to the one-dimensional scale.

This property makes dimensionality reduction using a single Peano curve practically inapplicable. To overcome this drawback, various approaches have been proposed, for example, the simultaneous use of two or more space-filling curves of different types [22], i.e., data shifting [9]. In the following section, we propose a constructive way to preserve some information on object proximity during dimensionality reduction based on the use of multiple same-type evolvents.

## 4. Constructing a Family of Peano Curves

Consider a family of Peano curves

(5) $$Y_{L}\left(x\right)=\left\{y^{0}\left(x\right),y^{1}\left(x\right),...,y^{L}\left(x\right)\right\}$$

instead of a single curve y(x). We will construct the family of curves as follows. Let us introduce a family of hypercubes

(6) $$D_{i}=\left\{y\in R^{N}:-2^{-1}\leq y_{i}+2^{-l}\leq 3\cdot 2^{-1},1\leq i\leq N\right\},\;0\leq l\leq L,$$

where the hypercube $D_{l+1}$ is obtained by shifting the hypercube $D_{l}$ along the main diagonal by step $2^{-l}$ for each coordinate.

Let a Peano-curve-type mapping $y^{0}\left(x\right)$ map the interval [0,1] onto the hypercube $D_{0}$ of (6), i.e.,

(7) $$D_{0}=\left\{y^{0}\left(x\right):x\in \left[0,1\right]\right\}.$$

**Figure 2:**
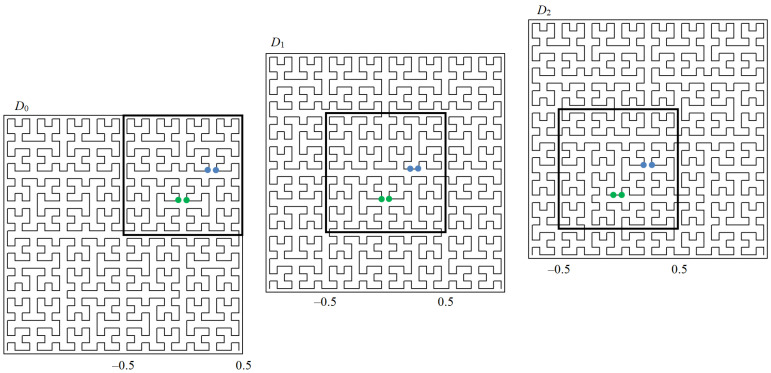
Family of Peano curves.

Any subcube of the *M*-th partition of the hypercube $D_{0}$ will have an edge of length $2^{-(M-1)}$ and will be denoted by $D_{0}\left(M,v\right)$, where *v* is the left boundary point of the subinterval d(M,v) corresponding to this subcube. Then, the evolvents $y^{l}\left(x\right)=\left\{y_{1}^{l}\left(x\right),...,y_{N}^{l}\left(x\right)\right\}$, whose coordinates are determined by the conditions

(8) $$y_{i}^{l}\left(x\right)=y_{i}^{l-1}\left(x\right)+2^{-l},\;1\leq i\leq N,\;1\leq l\leq L,$$

map the interval [0,1] onto the corresponding hypercubes $D_{l},\;1\leq l\leq L$ (the broken line in Figure 2 shows the image of the interval [0,1], which is obtained by using the evolvent $y^{0}\left(x\right),x\in \left[0,1\right]$).

For any subcube $D_{0}\left(M,v\right)$ of the *M*-th partition of the hypercube $D_{0}$, there will exist a subcube $D_{l}\left(M,v\right)$ of the *M*-th partition of the hypercube $D_{l}$, and $D_{l}\left(M,v\right)$ can be obtained by shifting $D_{0}\left(M,v\right)$ along the main diagonal by the distance of

$$2^{-1}+2^{-2}+...+2^{-l}.$$

It follows from Equations (7) and (8) that if an interval d(M,v) is mapped onto the subcube $D_{0}\left(M,v\right)$, there exists a family of subcubes

$$D_{l}\left(M,v\right)=y^{l}\left(d\left(M,v\right)\right),1\leq l\leq L,$$

connected with the corresponding subintervals $d_{l}\left(M,v_{l}\right)\subset \left[0,1\right]$, where $d\left(M,v\right)=d_{0}\left(M,v_{0}\right),$$v_{0}=v$, such that

$$D_{l}\left(M,v\right)=y^{l}\left(d_{l}\left(M,v\right)\right),1\leq l\leq L.$$

Since the hypercube *D* from (3) belongs to the common part of the family of hypercubes (6) (the boundary of the hypercube *D* is highlighted in Figure 2), by then introducing an additional function

$$g_{0}\left(y\right)=\max\{|y_{i}\left|-2^{-1}:\;1\leq i\leq N\right\},$$

the original hypercube *D* can be represented as

$$D=\{y^{l}\left(x\right):x\in \left[0,1\right],\;g_{0}\left(y^{l}\left(x\right)\right)\leq 0\},\;0\leq l\leq L,$$

i.e., $g_{0}\left(y\right)\leq 0$, if y∈D, and $g_{0}\left(y\right)>0$ otherwise. Hence, any point y∈D has its preimage $x^{l}\in \left[0,1\right]$ at every mapping $y^{l}\left(x\right),\;0\leq l\leq L$, i.e.,

$$y=y^{l}\left(x^{l}\right),\;x^{l}\in \left[0,1\right],\;0\leq l\leq L.$$

The algorithmic complexity of computing all preimages $x^{l}\in \left[0,1\right],\;0\leq l\leq L,$ depends on the dimensionality *N*, on the partitioning level *M*, on the number of mappings *L*, and is O(M·N·L).

By using multiple mappings $y^{l}\left(x\right),\;0\leq l\leq L$, the following relationship between neighbourhoods on one-dimensional scales and neighbourhoods in the original multidimensional domain is defined.

**Theorem** **1.** Let $y^{*}$ be an arbitrary point from the domain D belonging to the interval with endpoints $y^{\prime },y^{\prime \prime }\in D$, which differ in the values of the only coordinate, and let

(9) $$|y_{j}^{\prime }-y_{j}^{\prime \prime }|\leq 2^{-p},\;y_{i}^{\prime }=y_{i}^{\prime \prime }=y_{i}^{*},\;1\leq i\leq N,i\neq j,$$

where p is an integer, 1≤p≤L−1, and j is the number of coordinate whose values for points $y^{*},y^{\prime },y^{\prime \prime }$ are different. Then, there exists at least one correspondence $y^{l}\left(x\right),0\leq l\leq L$, and preimages $x^{*},x^{\prime },x^{\prime \prime }\in \left[0,1\right]$ such that

$$y^{*}=y^{l}\left(x^{*}\right),y^{\prime }=y^{l}\left(x^{\prime }\right),y^{\prime \prime }=y^{l}\left(x^{\prime \prime }\right),\max\{|x^{\prime }-x^{*}\left|,\right|x^{\prime \prime }-x^{*}\left|,\right|x^{\prime }-x^{\prime \prime }\left|\right\}\leq 2^{-pN}.$$

**Remark** **1.** The conditions of the theorem distinguish a specific neighbourhood of the point $y^{*}$. This neighbourhood comprises only such neighbours of this point that can be obtained by shifting $y^{*}$ parallel to one of the coordinate axes by a distance not exceeding $2^{-p}$. By varying the value of j,1≤j≤N, under the conditions of the theorem, one can identify the nearest neighbours of point $y^{*}$ along N coordinate directions. According to the statement, the proximity of points in the N-dimensional space in a particular direction will be reflected by the proximity of their preimages on one of the one-dimensional segments. In this case, the corresponding one-dimensional intervals can be different for different directions.

The proof of this theorem is given in [20].

As an illustration, Figure 2 shows a family of space-filling curves in extended domains $D_{l}$, where a square with a dark border highlights the hypercube *D* belonging to each extended domain. The dots indicate proximate objects in the domain *D*, the distance between which will vary depending on the one-dimensional scale used. The effect of maintaining distance proximity on one of the scales is clearly visible. For example, objects marked with green dots will be far away on the first two one-dimensional scales and close to each other on the third one. Objects marked in blue will be far away on the first and third scales and close to each other on the second scale.

As mentioned above, instead of the theoretical Hilbert curve y(x), the evolvent $y^{c}\left(x\right)$, which is an approximation to y(x) with an accuracy of the order $2^{-(M+1)}$, determined by the relation (4), is used in practice. In this case, the parameter *M* can be chosen individually for each problem to be solved, based on the accuracy of the input data representation.

For example, the data in the DS-Skin dataset (Section 6.2) are pixel coordinates of an image with a resolution of 256 by 256. When mapping data to the standard hypercube *D* from (3), the coordinate distance between two points cannot be less than 1/256. In this case, using the parameter M=10 to construct the evolvent will be sufficient. If we assign numerical values to a small number of categorical features (as was done for the CarEvaluation dataset, see Section 6.2), the  coordinate distance between the features will be large; so, the value of *M* can be reduced (in our experiments, we used M=7).

The maximum number of simultaneous mappings in the set $Y_{L}\left(x\right)$ from (5) will also be determined by the accuracy of the input data, which affects the accuracy of constructing the evolvents $y^{i}\left(x\right)$. Let us assume that each of the mappings $y^{i}\left(x\right)$ is constructed using the same value of *M*; then, the value of *L* must satisfy the inequality L<M. Otherwise, the points $y^{\prime }=y^{l}\left(x^{\prime }\right),\;y^{\prime \prime }=y^{l}\left(x^{\prime \prime }\right)$ from condition (9) will correspond to the centre of the same subcube with p≥M, i.e., the equality $y^{\prime }=y^{\prime \prime }$ will be fulfilled, leading to violation of the conditions of Theorem 1.

## 5. Implementation Features of the kNN Method Using Multiple Space-Filling Curves

The scheme of implementation of the kNN algorithm using multiple Peano curves will look as follows. For every object $\omega_{i}$, a set of its preimages on different one-dimensional scales is assigned

$$\omega_{i}\to \left\{x_{i}^{0},x_{i}^{1},...,x_{i}^{L}\right\},$$

where l,0≤l≤L is the number of the one-dimensional interval.

The degree of proximity of a test object $\omega^{*}$ to one of the known objects $\omega_{i}$ can be expressed in terms of the distance

$$d_{i}^{l}=\left|x_{i}^{l}-x^{*l}\right|$$

on the *l*-th scale.

Thus, the distance $d(\omega_{i},\omega^{*})$ between two objects $\omega_{i}$ and $\omega^{*}$ on a one-dimensional scale will correspond to the minimum distance among all the mappings

$$d\left(\omega_{i},\omega^{*}\right)=\min_{0\leq l\leq L}d_{i}^{l}=\min_{0\leq l\leq L}\left|x_{i}^{l}-x^{*l}\right|.$$

If we find the minimum distance

$$d_{min}=\min_{1\leq i\leq n}d\left(\omega_{i},\omega^{*}\right),$$

we obtain the nearest neighbour.

Next, the search procedure needs to be run as many times as there are nearest neighbours to be identified. The main problem is that an object previously found on one scale can be found again, including on another scale. This requires either removing the values of *x* found objects from all scales or searching for *K* nearest neighbours within each scale and then merging the results.

The procedure of finding *K* nearest neighbours using multiple space-filling curves was implemented in two ways. The first of them (denoted hereafter as Algorithm 1) involves searching for *K* neighbours on each scale and then merging the results and selecting *K* neighbours from all scales. The peculiarity of this algorithm is that there is no procedure to remove the objects found from the scales.

The second way (Algorithm 2) involves finding the nearest object to the test object on each scale, determining the nearest object among those found, and then removing the selected object from all scales. In this case, the search for the next nearest neighbour is performed by repeatedly calling the same procedure. After all nearest neighbours are determined, the previously excluded objects are returned to their places and the algorithm is ready to work with the next test object.

Here, we present a more detailed description of these algorithms.

### Data Preparation

To run Algorithms 1 and 2, we need to compute the preimages $\left\{x_{i}^{0},x_{i}^{1},...,x_{i}^{L}\right\}$ on one-dimensional scales for all objects $\omega_{i}$, and order them in ascending order. It is convenient to use RB-tree-based data structures to store ordered one-dimensional values, where the algorithmic complexity of the search operation has the estimate O(log(n)). We used the container set from the standard C++ library as an implementation of the RB-tree. The number of instances of tree structures is equal to the number of scales.

The input data for kNN are the values of object features imported into the program from external files. A text label indicating the class to which the object belongs is read with each such object.

The input data of the algorithm also includes the feature vector $y^{*}$ corresponding to the test object $\omega^{*}$.
**Algorithm 1:** Procedure AFor each scale, search for *K* objects closest to $\omega^{*}$. The result of the search is a set of vectors of the nearest objects on each scale.The vectors of the objects found are inserted into an RB-tree according to the distance criterion $d_{min}$.After processing all the scales, the first *K* objects are extracted from the resulting tree and they are considered to be the search result.The number of objects belonging to each class is counted and the decision is made as to whether the test object belongs to a particular class.

**Algorithm 2:** Procedure B
For each scale, search for the closest object to $\omega^{*}$; the result is a set of distances between the test object and the closest object for each scale.The object with minimal distance on all scales is selected.This object is saved in a special vector and excluded from the set of objects.The whole procedure is repeated *K* times, resulting in a vector of *K* nearest neighbours.The number of objects belonging to each class is counted and the decision is made as to whether the test object belongs to a particular class.If reclassification is required, the procedure is called to recover the previously excluded objects and search for neighbours.


## 6. Experimental Results

Let us compare the kNN method that uses multiple evolvents (hereafter referred to as kNN-ME) with the kNN method using KD-tree (hereafter referred to as kNN-KD) [23] to handle multidimensional data.

The study was performed using several datasets, both randomly generated for testing and real-world datasets from the database https://archive.ics.uci.edu/ml/index.php (accessed on 25 May 2022):

1. DS-Random-1: a set of random data generated using the random module of the Python NumPy library. The data has a uniform distribution and contains values on an interval from 0 to 1.
2. DS-Random-2: a set of random data generated by the scikit-learn library (version 1.0.2) function sklearn.datasets.make_classification.
3. DS-SkinSegmentation: a dataset of dimension 3 (RGB values) to distinguish human skin colour from other objects in images [24].
4. DS-CarEvaluation: a dataset of dimension 6, representing data from a car valuation model based on the values of 6 features [25].
5. DS-WISDM: a dataset of dimension 3, representing accelerometer and gyroscope time-series sensor data collected from a smartphone and a smartwatch [26].

Two kinds of studies were carried out:

- The time required to find *K* nearest neighbours for a test object was measured;
- The accuracy of recognition of test objects was studied, based on the analysis as to whether their neighbours belong to certain classes.

We employed in our experiments the kNN-KD method from the scikit-learn library; this method was used with default settings. For our kNN-ME method, we used the evolvent density m=12; the number of evolvents simultaneously used was L=10 for datasets 1–3, L=7 for dataset 4, and L=8 for dataset 5.

The computational experiments were performed on the following test infrastructure (Table 1).

### 6.1. Investigation of Neighbour Search Times Using Test Datasets

Several datasets were synthesised to examine the search time for neighbours. First, DS-Random-1 and DS-Random-2 sets consisting of 100,000 and 10,000 objects, respectively, were generated.

When generating DS-Random-1, the standard random module from the Python NumPy library was used. The data dimension *N* varied from 2 to 4; the generated data had a uniform distribution on [0, 1].

To generate the DS-Random-2 set, the sklearn.datasets.make_classification function from the scikit-learn library (version 1.0.2) was used with the following parameters:

- n_samples = 10,000 (number of objects),
- n_features = *N* (dimensionality),
- n_redundant = 0 (number of redundant features),
- n_informative = N−1 (number of informative features),
- n_clusters_per_class = 1 (number of clusters per class of objects generated).

**Figure 3:**
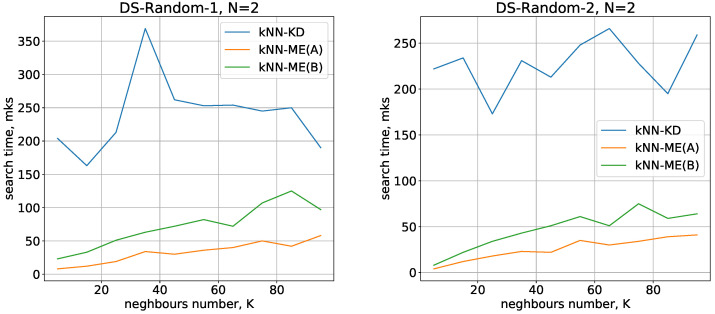
Running times of the algorithms with N=2.

The running times used by the different methods (kNN-ME, procedure A, kNN-ME, procedure B, kNN-KD) for DS-Random-1 and DS-Random-2 datasets with N=2 are shown in Figure 3. The abscissa axis represents the number of nearest neighbours that the algorithm is searching for; the ordinate axis represents the running time of the method.

**Figure 4:**
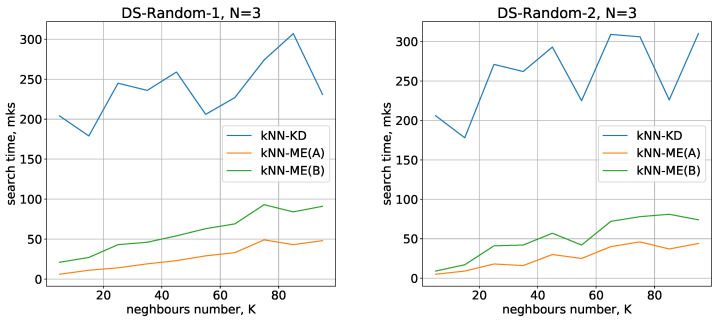
Running times of the algorithms with N=3.

**Figure 5:**
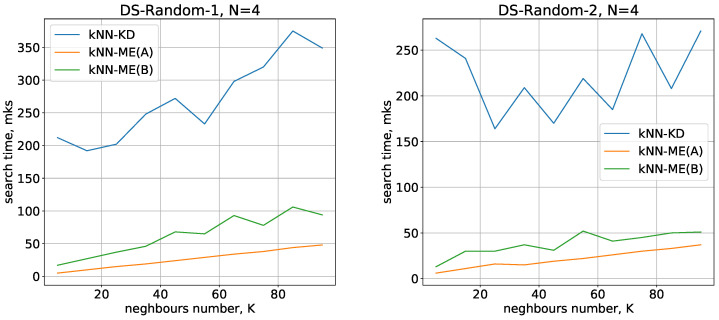
Running times of the algorithms with N=4.

Running times of the methods for nearest neighbours search with N=3,4 are given in Figure 4 and Figure 5, respectively.

All the methods considered show a small variation in running time when switching from one dimension to another. The number of objects being processed slightly increases the search time, and there is also a quasilinear increase in time as the number of neighbours identified increases. All these results are in good agreement with theoretical estimates of the time complexity of the methods under study.

On the whole, the experimental results show that the kNN-ME method based on search without removal (procedure A) works on average 2 times faster than the method that involves removal of elements found (procedure B). In fact, both variants of the kNN-ME method are 5–7 times faster (depending on the dataset) than the kNN-KD method.

**Figure 6:**
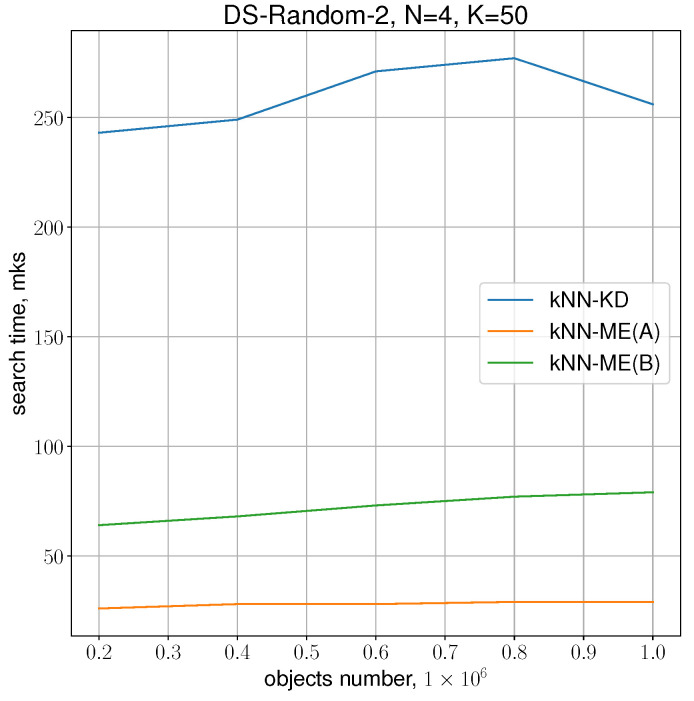
Running times of the algorithms with N=4, K=50.

The following experiment was conducted to determine the dependence of the search time for K=50 neighbours with increasing number of samples for the chosen dimensionality N=4. The number of samples was varied from 200 thousand to 1 million. The results presented in Figure 6 show that the difference between procedure A and procedure B remains approximately the same, and the gain of procedure A over the kNN-KD method is about 10 times.

### 6.2. Investigation of Neighbour Search Times on Real-World Data

Let us consider the DS-Skin set taken from the collection [24]. The objects in the set belong to two classes: the first class represents points in RGB colour space that belong to human skin images; the second class represents colour coordinates that do not belong to such images.

The number of objects of the first class is about 17,000, the number of objects of the second class is 43,000. The total number of objects examined is 60,000.

**Figure 7:**
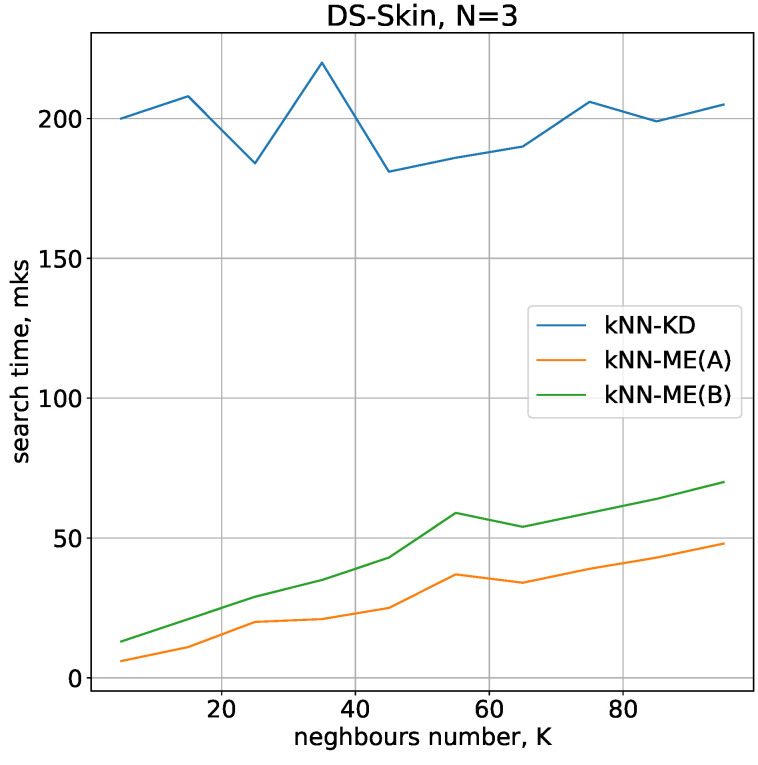
Comparison of neighbour search times when determining the colour of an image point.

The results of performance comparison of the methods on real-world data are qualitatively similar to those obtained by comparison on test data (see Figure 7). The kNN-ME method (implementation A) is 1.5 times faster than implementation B, and both implementations are at least 5 times faster than the kNN-KD method. Therefore, we will work with the kNN-ME(A) when investigating classification accuracy.

The performance of kNN-ME and kNN-KD methods was also tested using a real-world dataset [25]. There are 4 classes of objects in this dataset of dimension N=6. One particular feature of the dataset is that the numbers of objects belonging to each of the 4 classes differ widely (Table 2).

Further, note that this dataset will include several categorical attributes, each corresponding to 3–4 different values. In this case, it is necessary to assign numerical characteristics to discrete values. The numerical values used in the experiment were the internal nodes of a uniform grid covering the interval [0, 1]. The number of grid nodes was chosen to be equal to the number of feature values (3 or 4, respectively). Then, the *i*-th value of the categorical feature was assigned to the *i*-th node of the grid.

**Figure 8:**
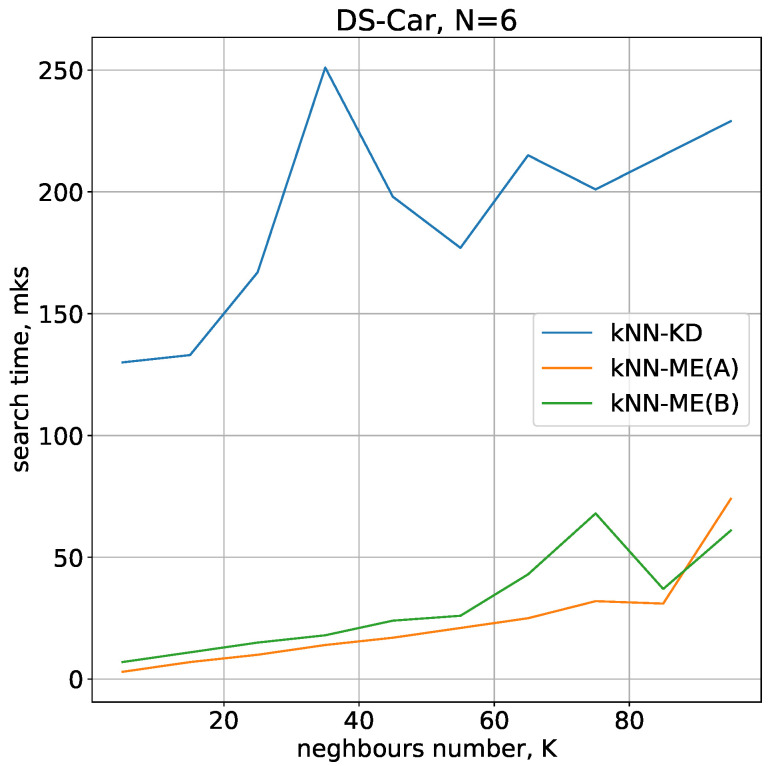
Comparison of neighbour search times for the CarEvaluation dataset.

The time required to find *K* nearest neighbours on the CarEvoluation dataset is shown in Figure 8. In this experiment, the number of evolvents used simultaneously was 7.

The results of the comparison on this dataset also demonstrate that the kNN-ME method is faster compared to the kNN-KD method.

It is of interest to study the dependence of the nearest-neighbour search time on the number of such neighbours in the case of a large number of objects. For this study, we chose the DS-WISDM dataset [26]. The raw accelerometer and gyroscope sensor data was collected from the smartphone and smartwatch at a rate of 20 Hz; it was collected from 51 test subjects as they performed 18 activities for 3 min apiece. The raw accelerometer and gyroscope sensor data were collected from a smartphone and a smartwatch at a rate of 20 Hz; it was collected from 51 test subjects as they performed 18 activities for 3 min apiece. Traditional use of this dataset involves raw data processing, which includes splitting the data into blocks of fixed size and calculating statistical characteristics for each block. Subsequently, each block is treated as an independent object with a set of inherent characteristics determining the subject’s activity during a given time interval. After such processing, the number of blocks will be substantially smaller than the original number of objects.

**Figure 9:**
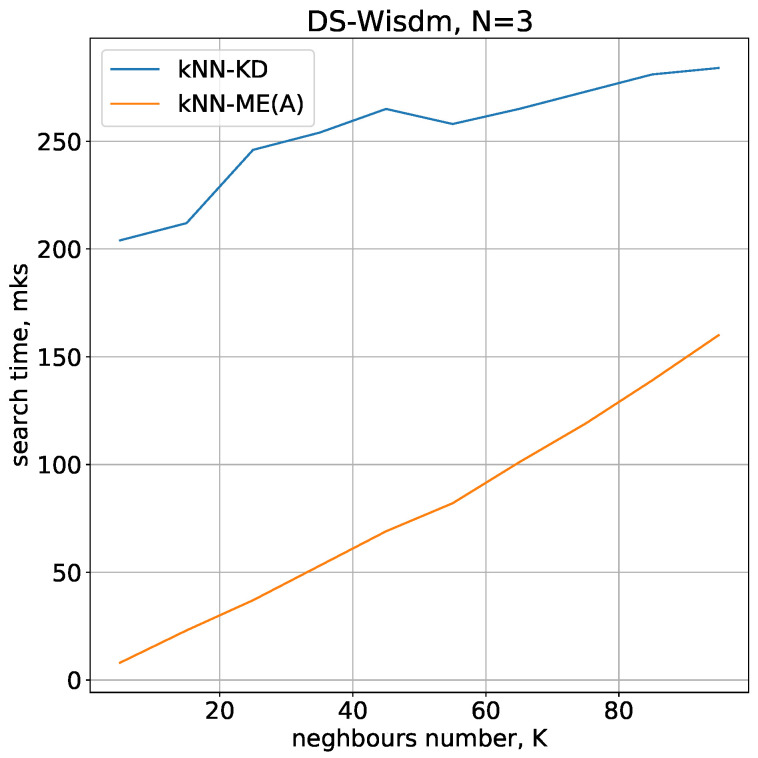
Running times of the algorithms with N=3, 7 million objects.

In our study, in order to keep a large number of objects in the dataset, we did not use this traditional approach and worked with raw data. Only spatial coordinates, with no time reference, were used to determine the nearest neighbours. Before conducting the computational experiment, we removed the duplicates found in the original dataset. After that, about 7 million objects belonging to 18 classes remained in the resulting set. Figure 9 shows the results of the nearest neighbour search in this dataset. Of our two neighbour-finding procedures, kNN-ME(A) was retained as a more efficient one compared with kNN-ME(B); the number of evolvents L=8 was used in this case. Similar to previous experiments, the results show that when the number of neighbours *K* is less than 100, the kNN-ME(A) procedure is also faster compared with the kNN-KD method.

The experimental results presented above show that even with the number of objects in millions, the running times of the algorithms considered in the paper remain small (within milliseconds). Therefore, the issues of parallelising and implementing multithreaded versions of the developed algorithms have not been considered in this paper.

### 6.3. Investigation of Classification Accuracy

According to the general idea of the kNN method, after finding *K* nearest neighbours to the test object, an attempt is made to determine the class of the test object. The more neighbours belong to a certain class, the higher the probability that the test object belongs to the same class. We will choose an odd number of neighbours, so as to avoid a situation where the nearest objects in equal numbers belong to different classes.

**Figure 10:**
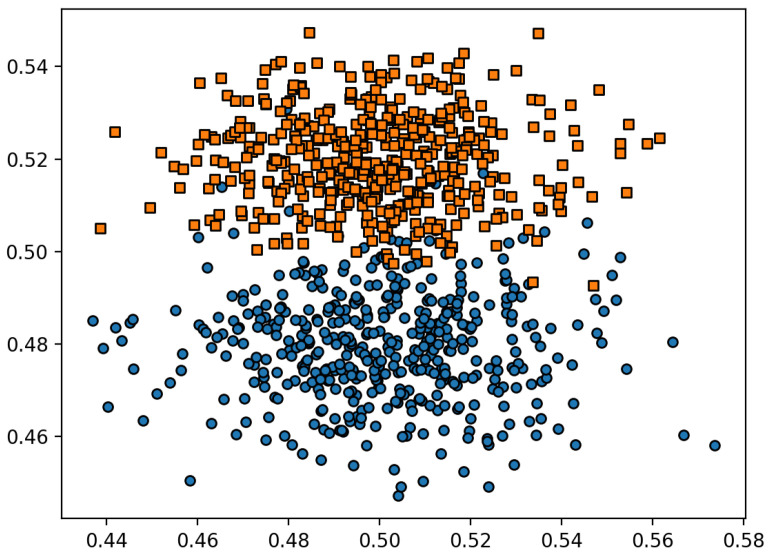
“Surrounded by aliens”.

Unfortunately, determining the class of the test object is prone to error. Errors may occur because of the “location” of the object under test on the hypercube edge in immediate proximity to objects of another class. Figure 10 shows an example of a random test set where some objects of one class are surrounded by objects of an “alien” class.

DS-Random-2 with N=2,3,4 and DS-Skin were used as datasets. The experiment to recognise the class of test objects was carried out as follows.

1. The source dataset was divided into two samples—a training sample and a test sample. The test sample size was 10% of the initial number of objects.
2. *K* neighbours were searched for each object in the test sample.
3. The class of an object under test was determined from the set of neighbours found.
4. The recognition result was checked against the known test object class and an error was recorded if there was a discrepancy with the result. The errors were summed up for the whole test sample.

To divide the source dataset into a training sample and a test sample, we used the sklearn.model_selection.train_test_split function from the scikit-learn library (version 1.0.2).

The results of investigating recognition errors on the synthetically generated random datasets (DS-Random-2) are given in Table 3. The numerical values in the table show the percentage of recorded recognition errors.

Based on the results of the experiment with random sets, we can conclude that the kNN-ME method has a recognition accuracy close to that of kNN-KD. The error rates of the methods differ, on average, by no more than 2–3%.

It should be noted that both kNN-ME and kNN-KD demonstrated high error rates, which can be explained by the fact that when generating random data, numerous objects at the class boundaries are generated that are classified incorrectly.

The results of measuring the recognition accuracy on the real-world DS-Skin dataset show that, on real-world data, both methods show the same percentage of misclassification. The specific error percentages obtained with different numbers of neighbours *K* are shown in Table 4.

Let us consider the results of determining the class of objects in the CarEvaluation set. For objects of classes 1 and 2, we generated test samples containing 20 objects, and for classes 3 and 4, samples containing 5 objects were generated. The kNN-ME and kNN-KD methods showed almost identical results: objects from the first two classes were identified with an average error not exceeding 5%, while objects of classes 3 and 4 were identified with an average error of 15%. The average recognition error rates for all classes are shown in Table 5.

## 7. Conclusions

The paper considers a classical machine learning method, the *k* nearest neighbours (kNN) method, which is widely used in machine learning practice and has been proven to perform very well because of its “explainability” (the decision regarding the choice of an object class can be clearly explained). The results of practical implementation of one of the kNN acceleration approaches based on dimensionality reduction using space-filling curves are presented.

An implementation of the kNN algorithm that uses multiple space-filling curves to better transfer the metric properties of multidimensional space to one-dimensional scales is proposed. Theoretical statements are presented about the preservation of the proximity property of objects in multidimensional space and on one of the one-dimensional scales when dimensionality reduction is performed.

The software implementation of the kNN method using multiple evolvents (kNN-ME) shows 5–7 times faster performance compared with the algorithm using kd-trees (kNN-KD). The comparison was conducted on test and real-world datasets of different size and dimensionality.

Both algorithms demonstrate comparable performance, measured as the percentage of misclassification of objects from the test sample. The number of neighbours in the classification was varied from 5 to 100. When applied to the synthesised random data, both kNN-ME and kNN-KD show a classification error rate of approximately 10%, while with real-world data both methods show an error rate of about 1%.

## Figures and Tables

**Table 1:** Test infrastructure.

Computer Specifications	
CPU	Intel Core i3-7100 (3.9 HHz)
RAM	8 GB
Operating system	Windows 10
Compiler	Intel(R) oneAPI DPC++/C++ Compiler, Version 2022.0.0

**Table 2:** Classes in the CarEvaluation set.

Object Class	Number of Objects in Class
1	1400
2	400
3	70
4	65

**Table 3:** Percentage of errors in DS-Random-2 recognition.

*K*	N=2		N=3		N=4	
	**kNN-KD**	**kNN-ME**	**kNN-KD**	**kNN-ME**	**kNN-KD**	**kNN-ME**
5	5	5	11	12	6	8
15	4	6	10	11	10	9
25	5	7	10	11	7	9
35	6	6	11	13	7	10
45	7	6	10	13	7	10

**Table 4:** Percentage of DS-Skin recognition errors, N=3.

*K*	kNN-KD	kNN-ME
5	0.5	0.5
15	0.5	0.5
25	0.5	0.5
35	1	1
45	1	1

**Table 5:** Average error rate (in percent) for DS-CarEvaluation recognition, N=6.

*K*	kNN-KD	kNN-ME
5	8	11
7	9	10
9	8	10
11	10	11
13	9	11

## Data Availability

The data analysed in this study are openly available at https://archive.ics.uci.edu/ml/index.php (accessed on 25 May 2022).
